# Thematic analysis of the raters’ experiences administering scales to assess depression and suicide in Arab schizophrenia patients

**DOI:** 10.1186/s12888-022-04313-3

**Published:** 2022-10-21

**Authors:** Iman Amro, Suhaila Ghuloum, Samer Hammoudeh, Yahya Hani, Arij Yehya, Hassen Al-Amin

**Affiliations:** 1 Department of Research, Weill Cornell Medicine – Qatar, Doha, Qatar; 2 Department of Psychiatry, Mental Health Hospital, Hamad Medical Corporation, Doha, Qatar; 3 Department of Psychiatry, Weill Cornell Medicine – Qatar, Education City, P.O. Box 24144, Doha, Qatar

**Keywords:** Cultural adaptation, Thematic analysis, Suicide, Depression, Schizophrenia, Arabs

## Abstract

**Background:**

This study aimed to enhance the cultural adaptation and training on administering the Arabic versions of the Calgary Depression Scale in Schizophrenia (CDSS) and The International Scale for Suicidal Thinking (ISST) to Arab schizophrenia patients in Doha, Qatar.

**Methods:**

We applied the qualitative thematic analysis of the focus group discussions with clinical research coordinators (CRCs). Five CRCs met with the principal investigator for two sessions; we transcribed the conversations and analyzed the content.

**Results:**

This study revealed one set of themes related to the scales themselves, like the role of the clinician-patient relationship during administration, the semantic variations in Arabic dialects, and the design of scales to assess suicide and differentiate between negative symptoms and depression. The other set of themes is relevant to the sociocultural domains of Muslim Arabs, covering religion, families’ roles, and stigma. It also covered the approaches to culturally sensitive issues like suicide, taboos in Islam, and the gender roles in Arab countries and their impact on the patients’ reports of their symptoms.

**Conclusions:**

Our results highlight several cultural and religious aspects to tackle when approaching schizophrenia patients through in-depth discussions and training to improve the validity of the assessment tools and treatment services.

**Supplementary Information:**

The online version contains supplementary material available at 10.1186/s12888-022-04313-3.

## Background

Schizophrenia is a chronic mental disorder with a lifetime prevalence of 0.7% of the worldwide population [1]. People with schizophrenia are at increased risk of having depression and suicide [2]. Depression is a common comorbidity in patients with schizophrenia and a well-known factor that affects their quality of life [3–5]. Depressive symptoms are associated with cognitive impairments, deterioration of psychosocial functions, extended hospitalization, and increased risk of suicide [6]. The reported rate of depression among schizophrenia patients is 25% [7–9]. The Calgary Depression Scale in Schizophrenia (CDSS) is a valid instrument for assessing depressive symptoms in the schizophrenia [10], independently of the negative or extrapyramidal symptoms [11]. CDSS has been adapted and validated in several countries and cultures [12–16]. We have already established the translation and cultural adaptation of the Arabic version of CDSS in Qatar’s Arab population [17].

Schizophrenia patients are also at increased risk of attempting suicide [18, 19], and an estimated 10% commit suicide during their lifetime [3, 20]. Universally, suicide is a behavior linked to different biopsychosocial factors [21] depending on ethnicity and culture. For instance, the sociodemographic and cultural factors for suicide in Asia differed from those in Western countries; however, there were no changes in the clinical characteristics [22]. The International Suicide Prevention Trial (InterSePT) Scale for Suicidal Thinking (ISST) consists of 12 items to assess the current suicidal ideation in patients with schizophrenia [23]. This tool was designed to study clozapine and olanzapine’s effects on suicidal behaviors in patients with schizophrenia or schizoaffective disorder [24]. The Arabic version of ISST has also been validated to assess the severity of suicidal ideation in Arab schizophrenia patients residing in Qatar [25].

Properly identifying the psychiatric signs and symptoms associated with schizophrenia is challenging [26], especially when using universal psychometric measurements and tools originated in a different cultural context. Researchers agree that psychiatric assessment depends on the clinical components and the subjective expressions related to the emotional states and the interpretations of the patients’ personal experiences and issues [27]. Subjective experiences are quite challenging to elicit from patients with mental health conditions who might have language and memory problems. However, there is evidence that people with mental health conditions can reliably and validly report their experiences [28, 29]. Culture can influence the various aspects of psychiatric illness like the presentation, behavior, assessment, severity of symptoms, and management [30, 31]. A recent study addressed the assessment bias of psychotic experiences in various countries. They concluded that such experiences are both under- and over-estimated in low- and middle-income countries compared to high-income ones [32]. A culture is a group of behavioral norms and values utilized by a particular community to construct their specific and unique perceptions of the world. Culture encompasses variables like language, traditions, religion, and many more [33]. In Arab countries, Arabs share cultural factors like religion, language, and peculiar beliefs and values, which might affect their subjective experiences with mental illness [34]. Culturally, how people perceive and deal with depressed patients differs according to their beliefs and cultural perspectives. Arab cultural beliefs and practices can be decisive in shaping Arabs’ perceptions and management of psychiatric disorders [35].

During the quantitative validation of the Arabic CDSS [17] and ISST [25], we undertook focus group discussions with the clinical research coordinators (CRCs) administering these scales. Focus group discussions are a useful and flexible qualitative method that provides information related to participants’ experiences [36, 37]. This paper presents the qualitative analysis of these discussions on the CRCs’ experience and the issues they encountered while administering the Arabic versions of ISST and CDSS for Arab schizophrenia patients. These focus group sessions and the analysis aimed to identify and tackle the relevant themes to improve the utility and validity of the Arabic CDSS and ISST in Arab populations.

## Methods

This study was part of a project to translate and culturally adapt scales used to assess patients with schizophrenia. We did the project in Qatar between 2013 and 2015, supported by Qatar National Research Fund (QNRF) (Grant number: NPRP 4–268–3-086). It was approved by the Institutional Review Boards of both Hamad Medical Cooperation (HMC) and Weill Cornell Medicine (WMC-Q) (Protocol number: 11129/11) in Doha, Qatar. These focus group sessions and qualitative studies were part of the raters’ preparation and training in administering the scales. All the subjects involved signed a written informed consent after explaining the study procedures’ details and duration. The scales were translated and validated to assess Arabic patients with schizophrenia. The information on the translation process, pilot study, and further details on the validation project methods are available in the published papers [17, 25].

### Study setting

Qatar is one of the Arabic Gulf countries in the Middle East, with an estimated population of 2.5 million, of which 28% are Arabs, including Qatari and non-Qatari [38]. Qatar is a rapidly developing country where most of the population are expatriates, but the Qataris and other Arabs are the most stable population. We conducted the study at the Mental Health Hospital, Department of Psychiatry, HMC, the main psychiatric facility in Qatar. The hospital has four inpatient wards (70 beds with 95% occupancy) and ten outpatient clinics with about 120 visits daily.

### Focus group participants and approach

We had two focus group sessions (90 minutes each) to discuss the Arabic versions of CDSS and ISST. We held discussions in a comfortable and familiar setting; We used both English and Arabic throughout the sessions. Members who participated in the focus group discussions comprised the CRC’s team and the study moderator who facilitated the discussions. All participants were bilingual, speaking English and Arabic. All the five CRCs were health professionals holding different medical backgrounds, including medical doctors, psychiatrists, clinical psychologists, and nursing, with varied experiences in mental health research, ranging from 3 to 5 years. They were first supervised on interviewing subjects and administering scales; each CRC was trained with 20 participants before the actual recruitment of subjects. The CRCs administered the Arabic CDSS to 109 subjects with schizophrenia and 102 controls, while Arabic ISST was administered to 110 schizophrenia participants and 99 controls. The study moderator used a semi-structured topic-guided approach (see the guide as a supplementary file) to discuss and explore the CRC’s feedback about the scales’ administration and address the barriers and issues they encountered in the recruitment process. We discussed all comments and feedback about patients’ responses as well.

We recorded and transcribed the discussions and took notes on specific questions and issues during the debate. The moderator adopted the semi-structured approach. He covered all aspects, including the recruitment process, CRCs’ experiences and encounters, and the scales themselves, by asking about individual items on each scale and the cultural and translational issues.

### Data analysis

Focus group discussions were recorded and subsequently transcribed manually using the English language. We used the thematic analysis approach [39] for this study: 1) We read the transcript notes several times to become familiar with the content and identify primary patterns. The notes were reviewed independently by the moderator and an independent reviewer for quality control and to ensure they were inclusive and comprehensive. 2) using these notes, we coded each item in the data repeatedly; we manually generated as many codes as possible during the coding process. 3) the initial codes and items were reviewed to identify the themes and subthemes. 4) all themes and subthemes were examined to check their relations and patterns. 5) Also, to ensure consistency and coherency, all topics were tested against each other and the original data. Accordingly, some themes were broken into smaller components, while others emerged together as the main themes. During the analysis process, continuous monitoring was done to provide a clear, convincing, and well-organized representation of the data and the topic. 6) Later, all quotes from the group interactions were listed for each theme in a narrative way where appropriate. We did revisions for the final items to ensure we identified the issues related to the available data. Other team members who were not involved in the translation or focus group discussions were also involved in the analysis to reduce bias and to confirm that we thoroughly analyzed the data and that the themes were consistent and reflective of the discussions. We kept the data confidential to ensure participants’ anonymity by changing all identification data into unique identifiers during the analysis process. However, quotes in the results section appear without any identifiers.

## Results

We will present the themes from the thematic analysis in two sections: 1) those related to the translation, cultural adaptation, and administration of Arabic CDSS and ISST, and 2) themes associated with the Arab and Qatari cultures.

### Items related to scales’ design and administration

#### Design of ISST

One central point related to the ISST design is the lack of instructions on delivering the questions. The CRC commented on this point: “when assessing the risk of suicide, the first question asks if the patient has any suicidal thoughts. If the patient answered, NO, I have no suicidal thoughts, you still have to ask the following questions, which are all designed to elaborate more on suicidal thoughts.” This series of questions will increase the risk of provoking anxiety in the patient. Also, there are no prompt or explicit instructions to tell the CRCs when to skip the following question or to continue when appropriate. So, the team thought that having clear guidance would save time and avoid asking unnecessary items that could impact the patient’s cooperation in completing the questionnaires.

CRCs discussed the time frame of the patients’ suicidal thoughts. The team questioned the seven-day criteria linked to this scale; they raised the scenario in which a patient had suicidal thoughts 10–15 days ago but not lately, for instance, wondering if such patients would be automatically excluded from the study. However, this is not the case in the CDSS, as it has clear instructions to prepare the CRC and the patient for the next questions. “As per the instructions in CDSS, we do not have to read all the potential answers in advance. We ask, wait, and then we elaborate to get the right answer; hence, it is the answer based on the assessment more than giving scores.” Another CRC commented on the differences in time frames in the two scales when assessing suicide: “using two different scales to assess the same element with two different time frames will affect the reliability of the patients’ responses, which might affect the rating scores. So, we should clarify this to the patient … “.

#### CDSS, self-rating vs. rater’s assessment

The group discussed the differences between the self-rating scales vs. those based on raters’ assessment. They agreed, “It is a lot easier to get the answers from the patients by reading all the potential answers among all the scales, and the patients will pick what suits them.” Also, they elaborated that with more explanation and re-wording, the final rating will depend on the assessment of each rater, which might introduce more discrepancies among the CRCs’ scores.

The group agreed that CDSS is designed to be used by trained raters and not intended for self-assessment. Thus, the best approach is to follow the standard procedure to administer the CDSS, where they read the answers, and then patients can pick their choices without their interference to avoid affecting the final score. However, one CRC commented that: “because it is based on your assessment, again you might have to make explanations, and depending on the answers you decide where they fit; the answer is not yes or no... You have to use your assessment, especially in the last item, as it completely depends on your assessment, not on what the patient says.”

#### CDSS, depression vs. negative symptoms

The group elaborated on how and what in the CDSS to differentiate depressive symptoms from negative symptoms of schizophrenia. Examples of questions used to facilitate the discussion included: “Are you able to differentiate, by using CDSS, between depression and negative symptoms? Do the questions help to differentiate these two entities, or do they still look the same for you?” After significant deliberation and guidance from the PI, the group agreed on the following points:

1. “It is very challenging to differentiate between negative symptoms and depression.”
2. “The concepts overlap a lot; in depression, the patient could be agitated or passive; he could be sleeping a lot or sleepless; he could be anxious or blocked. The challenge is that depressed patients might feel hopeless and always bored; everything they do is wrong, and they wish to die. But schizophrenia patients with negative symptoms don’t say such things; they will say nothing; if you ask him, he will say, I’m okay, everything is good, and you barely get yes or no answers from them. Even though they both might have a flat affect, the core is different.”
3. “Schizophrenia patients suffering from negative symptoms would not talk about depression even if they both look the same. You cannot get him to elaborate …. ”
4. “It is challenging, patients with schizophrenia might get depression separate from the schizophrenia, which will predispose them to suicide ….”
5. “Depressed person is the person who changed from a productive person to someone who is not, while someone experiencing negative symptoms (like alogia, no motivation, no initiative) will be different … will always be like that, and most do not recover...”

### Sensitive issues in Arab culture

One CRC said, “The scales are culturally sensitive …” and he suggested “spending more time at the beginning of the interview to explain it and to know how you will enter the grey area, this will probably help the patient to feel more comfortable to answer the questions … meaning to prepare the patient for the upcoming questions … and I’m talking about this and that … etc., so that could probably prepare them to the culturally-sensitive matters …” .

#### Rapport with patients

CRCs discussed the patient-clinician relationship and how that could be essential in developing a connection with the patient. All agreed that in the Arab culture, doctors have the authority to ask, discuss, and get answers from the patients much more comfortably than the CRCs do. “In the clinical setting, if you are a doctor, you have the authority; for example, the patient thinks, “I should tell the truth to the doctor and be honest and say what’s on my mind ….” They did not feel that this applies to their relationship with patients. However, CRCs pointed out that training and expertise have helped them to quickly develop a rapport with the patients, considering the amount of time they have. So, the question was raised by one of the CRC “...Can we establish rapport within five minutes with the patient? … “ The group agreed that “… the idea is not to jump and start into the topic if you feel that the patient is not comfortable and relaxed, but yes, he will not trust you and tell you all about his life; also, you need to know that even in the clinical setting, you can’t afford more than this time to do so ….”

#### Suicide and religion

ISST was designed to assess suicide among schizophrenia patients. However, suicide is a taboo within the Muslim community. As the subjects were Arabs and Muslims, the CRCs repeated: “It is very challenging to open and discuss this issue with the participants.” In response to the question: “Have you ever felt that talking about suicidality is culturally sensitive?” One CRC replied, “… yes, of course, and the patients are mostly upset … and try to convince us it’s not good, and we are not allowed to talk about it. They were always saying phrases related to religion that it’s completely forbidden … reacting defensively.” Another participant added, “I can’t recall someone super comfortable talking about this; even in the control group, they flip when you ask them about suicidality, it’s always a closed subject for them.”

Another interesting point to mention is “… even if they experienced this at some point in their life, and although they were admitted with suicidal thoughts, if you ask them, they will say no, so, at this point, you can’t ask and tell them no it is okay let’s talk about it.” Accordingly, the moderator asked, “Did you feel that the patients were hiding the reality, or were they genuine in their answers?” The answer was, “Well, some of them hide, and others are genuine, but down the line, you ask them several questions about the suicidality, and eventually, the reality will come out. I do not think they are comfortable talking about this …. “.

Also, question 8 in ISST covering the deterrents against attempting suicide might complicate the assessment further as “… we still have to explain it by mentioning religion or family as possible deterrents.” However, to minimize the cultural sensitivity, the group discussed the approaches to assess suicide in Arab culture without making the patients feel guilty and focusing on their suffering and how the disease can alter how we think and act on things. The group agreed that building rapport and addressing this matter using a neutral approach might help the rater’s assessment of suicide risk in this population.

CRCs pointed out that Arab and Muslim patients with mental illnesses mostly seek religious healers for blessing and spiritual support. The mental health professional is their last stop to seek professional advice. They confirmed that building rapport and spending more time with subjects was productive, especially when introducing the issue of suicide in a non-threatening and non-judgmental way.

#### Language- dialects and cultural influence

CRCs discussed the difficulties they encountered during the scales’ administration in terms of language. The spoken Arabic language has many dialects depending on the country of origin. It is common to find one word with several meanings, but “… more so in the various Arabic dialects ….” The formal Arabic language is not the same as the spoken dialect in each Arab country. Words can have one meaning in a dialect and have a different sense in another, which makes choosing one form of Arabic translation virtually impossible. Therefore, using the classical “formal” state of the Arabic language was the only way forward to overcome this issue; please refer to (Hammoudeh et al., 2016) [25] for more details about the translation process used for the scales. The classical form of the Arabic language is widely shared, understood, and used on all legal documentation in the Arab world; it was, therefore, easy for our CRCs to modify the written translated scales into acceptable and straightforward forms when interviewing the patients.

While interviewing the patients, translating some terms was challenging for the CRCs and the patients. For example, the translation for item 4 in the ISST, “passive suicidal ideation” in the Arabic language, was not very clear: “we always have to explain it more and give more detailed examples.” Also, in item 7, about “Delusions,” patients would understand this word differently; more explanation was always required to provide the right meaning to make it clear for the patient because, in Arabic dialects, there is no one translation for this word. An explanation of having delusions was needed; this is where the importance of receiving professional training was highly emphasized and recommended.

#### Mental illness and stigma

The team commented on the stigma in the Arab world, particularly in the Middle East countries where mental health illness is profoundly affected by the culture and the local norms. For example, “… family interference plays a significant role in this region where most of the mental illness cases are not properly managed, simply to avoid being labeled as a psychiatric patient.” We extensively discussed stigma as it has affected the recruitment process from the CRC’s point of view. One CRC said, “ … I have come across this situation more than once, where the patient refused to come for a follow-up interview in the validation project because they want to avoid being near the psychiatric hospital and spotted by someone they know. They always request to meet at the general hospital …” The female CRCs added that this social stigma is more so for women with mental illness where “this stigma is aggravated by the well-known gender disparity in the Arab culture.”

## Discussion

This qualitative study utilized the thematic analysis approach to identify the relevant items encountered by the raters administering the Arabic versions of ISST and CDSS during the cultural adaptation of these scales to the Arab population residing in Qatar. Briefly, there are themes related to the scales’ original design, the differences between scales rated by patients vs. those based on raters’ assessment, and the differentiation between negative symptoms vs. depression by CDSS. The other themes are mostly related to the Arab and Islamic cultures: the role of religion and its impact on the assessment of suicide, the various Arabic dialects used by Arabs and their influence on the ratings, patient-clinician relationships in the Arab culture, and the intense stigma around mental illness among the Arabs especially for women. The translation, cultural adaptation, and validation of the Arabic ISST and CDSS have demonstrated that both scales are valid and reliable among Arab schizophrenia patients [17, 25]. To our knowledge, there are no qualitative studies on these scales in the Arab countries or other nations. Thus, we cannot directly compare our results with other similar studies. However, we will attempt in the next sections to discuss the social and cultural significance of these themes in the Arab or Islamic cultures and whether such themes or others have been reported when assessing suicide and depression in schizophrenia in other international studies.

We mostly addressed the significant issues related to scales’ design through the validation project’s training phase. The CRCs received professional training and regular supervision while administering the scales. We also held weekly meetings to discuss their concerns and unify the approach and skills during the recruitment stage, including handling the lack of instructions and questions, the barriers to rapport, and the disclosure regarding suicidality and depression. Concerning the CDSS efficiency in differentiating between negative symptoms and depression among schizophrenia patients, there was a general agreement that experience and professional training on the scales helped the CRCs master the skills and increased their knowledge about these two entities. It is vital for all staff working with schizophrenia patients to receive dedicated training in suicide risk assessment and management. Such training should emphasize the importance of addressing conditions that could be related to or overlapping with depression. This unified approach is crucial to ensure that the entire clinical team knows the associated risk factors and how to minimize them [18, 20]. Therefore, the best approach to address the issues related to the design and administration of these scales is to have extensive training and supervision early on concerning the relevance of rapport with subjects, clearing ambiguities in the structure of the scale, and proper differentiation of depression vs. negative symptoms in the Arab patients with schizophrenia.

Previous studies have highlighted the importance of cultural aspects when assessing symptoms of schizophrenia [40]. In this regard, reflections from the western countries showed the preponderance of depressive symptoms, delusions, and thought broadcasting and insertion, while non-Western countries showed more auditory and visual hallucinations [41, 42]. Further, a study assessing community care of patients with first-episode psychosis showed racial-ethnic differences. Non-Hispanic blacks showed higher scores on psychopathology and less provision of services when compared to non-Hispanic whites [43]. Thus, proper methods for cultural adaptation and translation of scales and qualitative research are essential to adapt scales that consider the social context and self-perception of symptoms [44]. In our study, the CRCs and patients emphasized that religion might play a role in minimizing the disclosure of suicide among schizophrenia patients because the Islamic religious beliefs are deeply rooted in their life, acts, and thoughts; these Islamic values forbid suicide [45]. The Arabic and Islamic values favor family and religious ties over individual needs or preferences. Thus, the act of suicide can affect the whole family or tribe [46, 47]. In general, within the Arabic cultural context, when the mental health symptoms become severe and noticeable to others, families try different kinds of help, usually through religious figures, local healers, and family members. Several months later, they seek help from mental health professionals [35, 48]. However, others suggested that family relations within the Muslim and Arab communities are powerful, impacting the treatment journey for the mental health patient positively [49]. When family relations are stable and supportive, patients who suffer from mental health problems often respond better to their treatment plans. The availability of families can enhance their emotional and economic support and practical support, for example, providing transport to attend appointments, which can improve the patients’ coping with their mental illness. These family-patient relationships are also relevant to the assessment and care of schizophrenia in many other cultures [50, 51].

Furthermore, studies have shown that religious values are protective factors among Muslims [52, 53]. A study comparing patients with schizophrenia from Hong Kong and Beijing also reported differences in the factors associated with suicide in each population. They concluded that the determinants of suicide attempts in these patients are significantly modulated by the sociocultural factors [54]. Another study reached the same conclusion comparing patients from the United States of America and India [55]. Another aspect of cultural adaptation is the Arabic language, characterized by wide varieties, in which the written standard differs substantially from the spoken vernacular [56]. Arabic speakers often manipulate what they say based on the context and setting, which can easily impact how patients express their clinical symptoms [57]. In general, CRCs reported that patients with schizophrenia could express their understanding of most questions on both scales. However, particular items needed more clarifications and explanations to get the right answer. Some questions addressed sensitive issues for Arab patients, like religion and suicide-related problems. Although the themes reported in this study appeared well known to the healthcare professional because of their knowledge and clinical experience, the published literature lacks documentation about the patients’ responses and perceptions about the assessment scales and the clinicians’ experiences. Other researchers also saw this aspect in other countries; for example, a study showed that the over-diagnosis of schizophrenia in African-Americans was mostly related to cultural and racial differences in assessing psychotic symptoms [58]. The clinician must understand patients’ identity and cultural background very well, as culture plays many different roles in the diagnostic process for any illness [59]. An international study focusing on the diagnosis of major depression and schizophrenia, using the International Classification of Diseases, recommended that professionals and caregivers should “take the emotional component of language, and the diversity of linguistic and cultural contexts, into account [60].” To overcome some of the semantic difficulties, the training and professional translation should adhere to the scales’ core meaning to minimize the impact of the different dialects and variations in the Arabic language.

## Limitations

This qualitative study had some limitations that are worth discussing. First, the various Arabic countries were not well represented by our sample of subjects and by the CRCs involved, and thus the findings might not be generalized to all Arabs. Therefore, individual studies from each Arab country and with local CRCs from the same country would reflect better the sub-cultures in each Arab country and enhance the clinical measurements in that population. Second, We did no individual interviews with patients or caregivers, and such qualitative data are essential to have the direct input of patients and their families. Such analyses would further understand the relevant cultural specifics when assessing and treating Arab patients with schizophrenia.

## Conclusions

Our findings in this qualitative study covered the CRCs’ experiences and the different cultural aspects of Qatar’s Arab population. The thematic analysis identified items related to the proper cultural adaptation of psychiatric scales and the importance of training in administering these scales. We should consider the sociocultural context during the early stages of training to help the raters and enhance the validity of the adapted instruments. The qualitative analysis also showed the importance of religion, cultural values, stigma, and social structure when assessing the psychotic symptoms and suicide in Arab patients with schizophrenia. Further, the lack of international research on the qualitative aspects of the variations and biases in the assessment tools of psychosis and depression in schizophrenia, our results support the need for more investigations on this topic. These matters should also be discussed early with the team, unifying the approach to handle them properly and improving the assessments with ISST and CDSS. Such qualitative methods with the raters, patients, and caregivers would guide psychiatry facilities and policymakers worldwide to serve schizophrenia patients better.

## Supplementary Information

**Additional file 1.**

## Data Availability

To protect the research participants’ privacy and confidentiality, our de-identified data are available only upon request and after compliance with the policies and procedures of WCM-Qatar, HMC, and QNRF for data sharing. Submissions can be submitted to researchcompliance@qatar-med.cornell.edu.

## Abbreviations

CDSS: Calgary Depression Scale in Schizophrenia
CRC: Clinical research coordinator
HMC: Hamad Medical Cooperation
QNRF: Qatar National Research Fund
ISST: The International Suicide Prevention Trial (InterSePT) Scale for Suicidal Thinking
WCM-Q: Weill Cornell Medicine in Qatar
