# Supplementary material for: Thematic analysis of the raters’ experiences administering scales to assess depression and suicide in Arab schizophrenia patients
Source: BMC Psychiatry. 2022 Oct 21;22:652. doi: 10.1186/s12888-022-04313-3 (PMC9587575; doi:10.1186/s12888-022-04313-3)
Supplement: Supplementary file 1 — Additional file 1. [file 12888_2022_4313_MOESM1_ESM.docx]

Appendix I: List of the questions used by the facilitator of the group discussions

1. What the group thought about the Arabic translations per se and did the patients comment on specific translational issues?
2. How did you handle the different Arabic sub-cultures when administering the Arabic CDSS and ISST?
3. What questions needed further elaborations or explanations during the interview?
4. What are the advantages and challenges encountered during the interview using the Arabic versions?
5. What themes or topics were difficult to engage patients with or to assess their responses?
6. When evaluating the patients, what cultural matters did you encounter and how did you handle them? What were the reactions of the patients to these matters?
7. When using the item descriptions in the scales, what challenges did you face?
8. When rating the different items, did you have to read all the descriptions of each rating after each assessment?
9. Did you rely on the records or the staff involved in patients’ care to finalize some of the items?
10. Did the training prepare you to handle the challenges you encountered during the assessment and rating procedures? Any suggestions for further training?
